# Animal Feed Formulation—Connecting Technologies to Build a Resilient and Sustainable System

**DOI:** 10.3390/ani14101497

**Published:** 2024-05-17

**Authors:** Oreofeoluwa Akintan, Kifle G. Gebremedhin, Daniel Dooyum Uyeh

**Affiliations:** 1 Department of Biosystems and Agricultural Engineering, Michigan State University, East Lansing, MI 48824, USA; oreofeakintan@gmail.com; 2 Department of Biological and Environmental Engineering, Cornell University, Ithaca, NY 14853, USA

**Keywords:** animal feed formulation, nutrition, livestock, decision support tools, environmental sustainability

## Abstract

**Simple Summary:**

In response to the challenges posed by a growing global population, the livestock industry must increase food production while ensuring environmental sustainability. This paper explores how feed producers can tackle these challenges using advanced formulation techniques. By leveraging AI decision support systems, producers can optimize feed composition to promote animal health and environmental stewardship. The study’s findings offer valuable insights into improving animal feed production, supporting the livestock industry in achieving sustainability goals and contributing to global environmental conservation efforts.

**Abstract:**

The unprecedented challenges presented by the increase in global population have placed substantial demands on the livestock industry for human nutrition, necessitating heightened animal productivity and leading to an increased demand for natural resources to produce animal feed. Feed producers are leading the charge, consistently refining formulations to adapt to the evolving needs of livestock, driven in part by the cost of over 50% associated with feed production. This paper critically analyses the pressing issues within feed formulation, addressing the requirement for environmentally sustainable practices amidst the challenges of climate change. The exploration extends to how advanced decision support tools can enhance formulation techniques and profitability and contribute to environmental sustainability. Through an in-depth review of current feed formulation technologies, encompassing their applications and limitations, this study aims to enhance the existing knowledge base. Additionally, we examined future trends, highlighting the essential role of connecting technologies to establish a resilient and sustainable system. The emphasis is on the potential of formulation techniques to positively impact the environment and enhance the overall quality and performance of the animals. This paper provides actionable insights to improve animal production by examining feed formulation models and decision support tools. The anticipated outcome is a more informed and sustainable decision-making process, addressing the multifaceted challenges confronted by the livestock industry and making contributions to global efforts in climate change mitigation and environmental stewardship in animal production agriculture.

## 1. Introduction

The global demand for high-quality animal products, driven by an increasing population and evolving dietary preferences, has cast an unparalleled spotlight on the livestock industry. Within this dynamic landscape, the formulation of nutritionally balanced and sustainable animal feeds emerges as the requirement for producing outstanding animal products that meet the demands of a changing world [1]. The West African region, comprising countries like Benin, Burkina Faso, Ghana, Nigeria, and Senegal, grapples with a surging population projected to exceed 800 million by 2050 [2]. This growth intensifies the need for food security, particularly in livestock farming, which is important for the region’s economy. Livestock contributes significantly to agricultural GDP, with up to 34% of rural incomes reliant on it. Despite abundant livestock diversity, meeting the escalating demand for animal products, notably red meat, poses a challenge. To address this, there is a critical need for improved production practices and policies to bolster local capacity. West Africa faces significant challenges and opportunities in its livestock sector. Enhancing feed formulation and sustainable farming practices is crucial for ensuring food security and economic growth amidst a changing world. As the 21st century unfolds, the obligations of ensuring food security, mitigating environmental impacts, and addressing the ethical dimensions of animal agriculture become ever more pressing. In response to these multifaceted challenges, the integration of cutting-edge technologies in animal feed formulation takes center stage, offering a pivotal pathway toward building a resilient and sustainable system [3]. Conventionally, animal feed formulation has been a process of intricately optimizing nutrient content to align with the specific requirements of diverse animal species and varied production goals [4]. The foundation of these formulations leaned heavily on nutritionists’ expertise, grounded in empirical knowledge and general guidelines. However, modern technologies have signaled a shift, leading to tools that enable a more precise and efficient approach and align with sustainability needs [5]. Conventional feed formulation techniques, while historically significant, have several disadvantages. They often rely on fixed ingredient ratios, which can lead to nutritional imbalances if the quality of ingredients varies. These methods typically ignore the dynamic nature of ingredient prices and availability, potentially resulting in cost inefficiencies [6]. Moreover, conventional techniques may not account for the specific dietary needs of different animal breeds or life stages, potentially compromising animal health and productivity. The lack of precision in these methods can also contribute to environmental issues, such as nutrient runoff from over-supplementation. Lastly, they are less adaptable to incorporating alternative and novel ingredients, which can be a limitation in the face of global feed resource challenges [7].

A key technological advancement shaping the landscape of animal feed formulation is the integration of computational models and artificial intelligence [8,9]. This transformative pair facilitates the analysis of vast datasets, considering factors such as animal physiology, genetics, and environmental conditions. Applying machine learning algorithms allows researchers and nutritionists to determine intricate patterns within these datasets, leading to a unique level of precision in formulating feeds. This precision optimizes animal performance and minimizes resource utilization, aligning with the broader sustainability goals. Beyond artificial intelligence, the fusion of molecular biology and genomics marks a new era of personalized nutrition for livestock. By unraveling the genetic makeup of animals, it becomes possible to identify specific nutritional requirements based on individual variations. This customized approach enhances feed efficiency and contributes to the overall health and well-being of the animals, signaling a departure from reliance on additives and medications [10,11].

In addition to these technological advancements, incorporating sustainable sourcing and processing technologies in feed formulation is a foundation for building a resilient system. In response to escalating concerns about environmental degradation and resource depletion, there is a growing emphasis on alternative protein sources, such as insect meal and plant-based proteins [12]. This shift towards sustainable feed ingredients reduces the environmental footprint of animal agriculture and addresses the ethical considerations associated with conventional feed sources. The interconnectedness of technologies in animal feed formulation extends its influence beyond the laboratory to the farm level. Precision feeding, made possible through automated monitoring systems and sensor technologies, facilitates real-time adjustments to feed rations based on animals’ individual needs and behaviors. This optimizes production efficiency and minimizes waste, creating a more sustainable and economically viable animal agriculture system [13]. The convergence of cutting-edge technologies in animal feed formulation is a pivotal force in constructing a resilient and sustainable system capable of meeting the challenges of our era. By harnessing the power of artificial intelligence, genomics, and sustainable sourcing, the industry is poised to tailor nutrition to individual animal needs, optimize resource utilization, and mitigate the environmental impact of livestock production. As we navigate the path towards a more sustainable and ethical future for the livestock industry, integrating these technologies in animal feed formulation is an example of innovation, guiding the way towards coexistence with the demands of a growing global population [12,14].

The objectives of this study were as follows: (1) to conduct a comprehensive review and critically analyze the pressing issues in feed formulation, addressing the requirements for environmentally sustainable practices, and (2) to review current feed formulation technologies, encompassing their applications and limitations and (3) to connect animal feed formulation models with decision-support tools and integrate artificial intelligence (AI) to optimize processes in the context of a sustainable environment.

In this review paper, we comprehensively searched scholarly articles published from 1980 to the present (2024). Our focus was on animal feed formulation and related topics. Additionally, we thoroughly gathered literature relating to decision support systems (DSS) for animal feed formulation, with a specific emphasis on the role of artificial intelligence (AI) in enhancing sustainability in feed formulation practices. By synthesizing the selected literature, our objective was to determine starring themes and trends. We systematically organized these findings into cohesive sections to explain the intricate relationship between technological advancements and the development of robust and sustainable feed systems.

## 2. Relevant Works

### 2.1. Animal Feed Formuation

Feed formulation plays a crucial role in the livestock industry. It ensures that animals receive optimal nutrition, contributing to economic viability and environmental sustainability. This process meets a wide range of human needs, including food, employment, clothing, and income [15].

As the human population burgeons, the livestock sector faces escalating demands, notably in animal feed production. This surge in demand necessitates livestock production to vie for natural resources, notably land and water, while fortifying resilience against environmental, social, and political upheavals [16,17]. However, in regions like sub-Saharan Africa, escalating water scarcity, exacerbated by reduced rainfall due to climate change or heightened demand from alternative sources, poses a grave threat to water availability for livestock production [11]. This creates a need for more prudent use of water resources in livestock production. Frequent and severe weather phenomena such as storms and floods may also decrease feed availability. Both water scarcity and overabundance in the form of storms and floods may lead to reduced feed production capabilities and result in high feed costs [18].

Climate change poses significant challenges to animal nutrition and productivity, with implications for sustainability in animal agriculture [19]. Rising temperatures exacerbate animal heat stress, disrupting metabolic processes, appetite, and nutrient utilization. Heat-stressed animals may reduce feed intake, leading to inadequate nutrient intake. Moreover, increased energy expenditure to regulate body temperature during hot weather reduces energy available for growth, reproduction, and milk production [20,21]. Heat stress also alters mineral metabolism, leading to electrolyte imbalances, and degrades vitamins crucial for overall health [20].

The effects of climate change on animal productivity are profound. Heat stress reduces growth rates in young animals and negatively impacts reproductive efficiency, reducing conception rates, embryonic mortality, and lower sperm quality [19]. Dairy cows experience decreased milk yield during heat stress, while poultry, especially layers, lay fewer eggs during extreme heat, affecting egg quality. Heat-stressed animals may also have tougher meat due to altered muscle metabolism [20]. To address these challenges, technological solutions are crucial. Providing shade, proper ventilation, and cooling systems can mitigate heat stress while formulating diets with heat-resistant nutrients like electrolytes, antioxidants, and amino acids helps combat heat stress. Feed additives such as probiotics, prebiotics, and enzymes enhance nutrient utilization and gut health. Precision feeding technologies ensure animals receive optimal nutrients based on their specific needs, while genetic selection for heat tolerance and resilience is essential [21]. Adopting sustainable practices, leveraging technology, and prioritizing animal welfare are key strategies to mitigate the adverse effects of climate change on animal agriculture. By implementing these measures, we can ensure resilient livestock and poultry production systems while promoting environmental sustainability [20].

Due to the increasing demand placed on land resources by population growth and the needs of various sectors, the availability of livestock grazing lands is diminishing [22]. Feed availability and the costs for production are essential to the livestock industry in terms of profit and overall sustainability. An estimate of feeding showed a contribution of 60–80% of the variable costs of animal products, such as pork, milk, and poultry production [23,24,25]. Cost-effective feed rations are, therefore, essential for building resilience in the livestock industry. Feed costs can be managed effectively with knowledge of the vital nutrient requirements for every growth and reproduction stage of a livestock species and the related costs. According to Minson et al. [26] and Underwood [27], relaxation of some nutritional requirements that may not be vital for a particular growth stage can lead to significant reductions in feed costs while maintaining the animal’s performance. This was demonstrated by Uyeh et al. [28], using an interactive feed formulation approach based on an evolutionary algorithm.

Animal feed formulation has been defined as allocating feed ingredients that satisfy the minimum requirements of nutrients to achieve specific objectives or achieve the best animal performance regarding yield and weight gain [29,30]. This implies the main aim of feed production is to achieve the optimal composition of ingredients, which results in the desired outcomes when fed to the animal. On the other hand, feed materials are the carriers of nutrients, and feed formulation allows the measurement and combination of several ingredients needed to supply the nutrient requirements of an animal with the desired aim [31]. Also, feed formulation consists of multiple objectives, such as maximizing land use and minimizing feed cost while meeting the appropriate nutrient requirements [28].

Feed formulation techniques have gone through many evolutions. In the 1950s, time-consuming electromechanical machines were used. The 1980s and 1990s brought in the computerized linear programming method to maximize income while considering feed costs. By the turn of the next decade, non-linear programming models were introduced in feed formulation to optimize weight gain in animals and increase yield in products such as milk. Standard practices in the formulation of feed in animal diets are constant formulation, least-cost formulation, and fixed formulation [32].

Constant formulations focus on delivering a consistent amount of nutrient levels. They account for differences in the biological natural feed components and aim to minimize variations in nutrients. The formulation is fixed, involving the addition of a fixed level of feed ingredients and nutrients. This ensures the feed’s nutritional content remains constant, regardless of variations in the natural ingredients used. Least-cost formulations, on the other hand, prioritize cost-effectiveness. They involve substituting a higher-cost ingredient with a lower-cost one while still meeting the nutritional requirements of the feed. The goal is to obtain the least-cost formula, hence the name. This method is used for commercial purposes where cost efficiency is a significant factor. While both methods aim to provide nutritionally balanced feed, constant formulations prioritize consistency in nutrient levels, while least-cost formulations prioritize cost efficiency. The choice between these methods depends on the specific goals and constraints of the feed production operation.

Feed formulation comprises two broad techniques: the manual approach and mathematical linear, single/multiple programming methods [31]. The manual approach consists of the Pearson’s square method, trial and error method, simultaneous algebraic equations, and the two-by-two matrix technique. Pearson’s square method is used to calculate the percentage of two feed ingredients needed to meet the specific nutrient (crude protein, energy, crude fat) level in a mixture. For example, grains such as corn and soybean meal can be used to achieve the crude protein requirement of an animal. It is easy to use; however, the limitation of this technique is that it cannot be used for complex feed mixtures [33,34]. Simultaneous algebraic equations, on the other hand, can simulate two or more feed ingredients to obtain the best possible nutritional content [34]. With this method, the requirements for both protein and energy can be obtained in one mixture, unlike Pearson’s square method, which allows for only one nutrient requirement. The trial-and-error method is more prevalent in poultry, and the limitation is that it is tedious and time-consuming [34]. With the two-by-two matrix technique, two feed ingredients are used to solve a two-nutrient requirement problem, the two-by-two matrix is fixed, and a series of equations is calculated to find the solution to the problem [4].

The mathematical linear, single, or multiple programming methods include the single-linear programming approach, a frequent animal diet formulation technique [35]. Waugh [36] made the first attempt at the single-objective programming method to solve a feed-mix problem by optimizing the ration for livestock with a linear program. In linear programming, the model assumes the nutrient requirement as fixed; a few disadvantages stated by Lara and Romeo [37] include that the model’s constraints are rigid, can tackle only linear constraints, and can only operate on the objective function. They are also an efficient tool in the decision-making process by solving a series of linear and non-linear programs via interaction with decision-makers. Zhang and Roush [38] mentioned many advantages of using the multiple-objective programming method, including trade-offs in the decision-making process, concurrent action, and tractability. The linear programming method has been the most used mathematical method in the past decades. Its weakness is based on its reliance on cost as the only basis for the decision-maker in feed formulation. These weaknesses can be remedied by decision-making techniques such as goal and multi-objective programming.

### 2.2. Decision Support Tools in Livestock Feed Formulation

Decision support tools (DSTs) play a crucial role in Livestock Feed Formulation (LFF) by addressing challenges related to diverse data sets and the complexity of monitoring animal-related systems [39]. These tools are essential for achieving two main objectives: improving animal welfare and mitigating greenhouse gas (GHG) emissions. Enhanced animal welfare directly impacts outcomes in livestock production, such as meat, milk, and eggs, as healthy animals yield higher quality and quantity of products. Additionally, optimizing animal welfare contributes to reducing emissions intensity in the production of livestock products, benefiting both farmers’ incomes and environmental sustainability [15,40]. Nutrition is a fundamental aspect of animal welfare, evaluated through the Five Domains Model, which emphasizes the importance of providing animals with appropriate feed and water [36,41]. Proper nutrition directly impacts animal health and well-being, influencing growth, reproduction, and immune function. Feed formulation plays a crucial role in determining the nutritional content of animal products, contributing to high-quality meat, milk, and eggs. Additionally, sustainable feed formulation practices promote natural resilience in animals, reduce the need for pharmacological treatments, and minimize environmental impact by reducing nutrient runoff. Prioritizing animal nutrition supports their welfare and ensures the production of safe, high-quality food for consumers, fostering a sustainable agricultural system [41].

Some existing designed decision support tools include diet checks. This method creates a way to estimate whether dairy cows are consuming enough crude protein, metabolized energy, and neutral detergent fiber for a stipulated milk production quantity [42]. Milgen et al. [43] designed and developed another model, the InraPorc tool, to be used by animal nutritionists to evaluate the utilization of nutrients and experiment with different nutritional strategies. Dobos et al. [44] also introduced the FeedPlan decision support tool, which combines parts of managing herd requirements and feed supply as a simple approach to feed planning and budgeting.

Decision support tools are developed to enable the appropriate users to make effective decisions via a clear description of precise steps to undertake and display the various results obtained from the several options executed [45,46]. These tools can be dynamic and non-dynamic software tools with different recommendations specific to the user’s inputs. The software tools can help achieve effective farm administration via efficient data recording, analysis, and production of a series of results based on the evidence gathered [47]. Decision support tools in feed formulation can be utilized by applying multiple criteria decision-making techniques. This approach has more advantages over the linear programming technique, such as simultaneously manipulating various conflicting objectives [48].

Rose, D.C. et al. [49] recommend taking into consideration the following factors in designing effective decision support and delivery tools: cost-effectiveness, habit, trust, ease of use, performance expectancy, peer recommendation, relevance to the user, farmer–adviser compatibility, facilitating conditions, information technology, age, the scale of farming, farming type, compliance, and levels of marketing, influencing the use of these tools by the farmers and advisers. The authors concluded that these factors mentioned above could serve as a checklist for a superior design of decision support tools in the future.

### 2.3. Techniques in Feed Formulation

Trends in feed production, such as advanced models for precision feed production, have the potential to boost livestock productivity and aid in dissipating the pressures it faces. Black, J.R. and Hlubik, J. [50] illustrated the basics of ration formulation via computerized linear programming, outlining the application, upper and lower bounds on feedstuffs, model ratio, and more. Using the linear programming method, a shrimp diet was formulated in Barbieri, M., Cuzon, G. [40]. Eight feed ingredients and eighteen constraints were used in diet formulation, and an optimal solution with a cost reduction of about 30% and no significant loss in the growth performance was achieved. More advanced computer techniques for feed production were developed in the ’90s. Lancaster, L.M. [51] developed software for mainframe computers as the foundation for computer-assisted menu planning. Another example is the multi-agent modeling approach Hamel et al. [42] applied to simulate vegetal raw material for poultry production chains. This model represents the multi-faceted workings of decision-making and human input in the feed formulation process. It operates in Java programming language, simulates the processes in production, and delivers the results. Table 1 shows the evolution of feed formulation techniques with the applied models from 1980 to 2020.

Over the past four decades, animal feed formulation has witnessed a notable evolution in the methods and models employed to optimize the intricate processes involved in meeting the nutritional needs of diverse livestock. The trend and prevalence of various techniques and models can be examined in Table 1 [76,83,91]. Beginning in 1980, the linear programming (LP) algorithm emerged as a common choice due to its versatility and applicability to a wide range of optimization problems. However, challenges persisted in accommodating the intricate degrees of feed formulation, particularly when faced with diverse nutritional requirements for different animal species and varying production objectives.

In response to these challenges, multi-goal programming and multi-objective optimization algorithms emerged as potential solutions in the 1980s and 1990s [38,69]. These methodologies allowed for the simultaneous consideration of multiple objectives, facilitating a balanced and holistic formulation that optimally catered to the diverse needs of various livestock. Furthermore, mixed-integer and non-linear programming techniques were explored to enhance the precision of feed formulation models, incorporating discrete variables and accommodating nonlinear relationships inherent in nutritional requirements and ingredient interactions. By the early 2000s, simulation became a powerful tool in animal feed formulation, creating virtual environments to model and analyze the impact of different feed formulations on animal health, growth, and overall performance. Integration of spreadsheets with other programming algorithms also gained traction, promoting efficient data manipulation and computation for practical application [40,57].

As the complexity of feed formulation continued to evolve, multi-criteria decision models and stochastic programming techniques were introduced to acknowledge and mitigate uncertainties associated with ingredient variations and environmental factors. Goal programming prioritized specific objectives while minimizing costs and aligning feed formulations with economic viability. In the latter part of the 2000s and into the 2010s [73], introducing linear fuzzy models and the proximal bundle approach added flexibility and adaptability to the formulation process, handling imprecise or fuzzy data more realistically. Real-coded genetic algorithms, drawing inspiration from natural selection and genetic mechanisms, offered an innovative means of optimizing feed compositions through iterative evolution. The challenges encountered in animal feed formulation, characterized by diverse nutritional requirements and fluctuating ingredient availability, have driven the exploration of advanced mathematical programming and artificial intelligence-driven solutions [89]. From linear programming in the early 1980s to evolutionary algorithms in the late 2010s, these diverse algorithms represent opportunities to transform feed formulation [50]. The fusion of AI technologies with mathematical models promises to overcome existing challenges, leading to more efficient, precise, and sustainable practices in animal nutrition management.

### 2.4. AI-Driven Solutions for Resilient and Sustainable Agriculture

**Figure 1:**
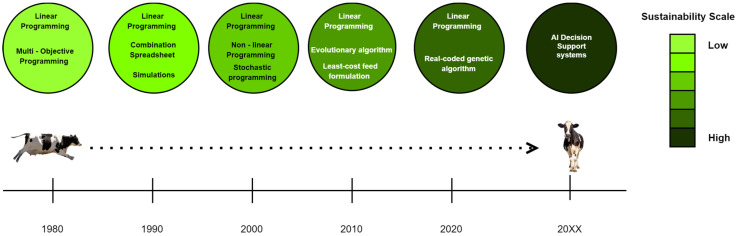
Moving towards AI decision support systems with the evolution of formulation methodologies over the decades.

AI-driven decision support systems offer a sustainable approach to resilient agriculture by revolutionizing feed formulation processes [76]. Optimization algorithms, such as linear programming and genetic algorithms, integrated into AI systems determine the optimal blend of ingredients in animal feed formulations. By considering factors like nutritional requirements, ingredient availability, cost constraints, and dietary regulations, these algorithms generate balanced and cost-effective feed compositions that promote sustainability in agriculture. Predictive models, powered by machine learning techniques, are crucial in selecting suitable feed compositions based on historical data on animal performance, feed composition, and environmental factors. By forecasting the effects of different feed formulations, these models aid in optimizing feed compositions for specific livestock breeds, growth stages, or production objectives [92]. This data-driven approach minimizes resource waste and ensures efficient use of ingredients, contributing to sustainable feed production [92,93]. Figure 1 illustrates the evolution of feed formulation techniques, leading to AI decision support systems that foster improved sustainability practices.

The knowledge base, serving as the basis for decision support systems (DSS), encompasses essential data, information, and knowledge required for informed decision-making. Modern knowledge bases facilitate the storage of both structured and unstructured data, aggregating information from diverse sources, including spreadsheets, data streams, external APIs, and sensors tracking and monitoring livestock. Furthermore, the knowledge base captures model analyses, simulations, and user feedback as valuable data points contributing to system enhancement in the future [7,94]. Operating within the context of animal feed formulation, the DSS model plays a crucial role in optimizing feed composition through either a simple rule-based system or a complex machine-learning algorithm. These models generate simulations and identify trends, patterns, and insights, providing recommendations relevant to animal nutrition [95].

**Figure 2:**
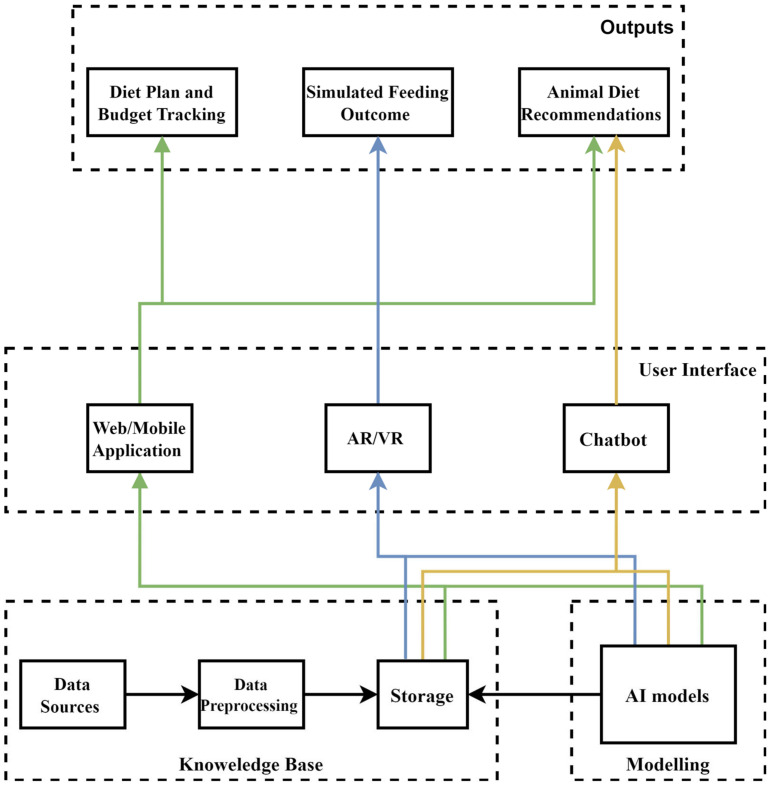
Connecting technologies to build a resilient and sustainable system (Line colors used to differentiate the User Interfaces).

The user interface of the decision support system incorporates advanced technologies to enhance user interaction and system capabilities. Natural Language Processing (NLP) powers the chat box feature, enabling conversational interactions and providing personalized diet recommendations based on user queries. Virtual Reality (VR) utilizes Generative Adversarial Networks (GANs) to simulate diet outcomes visually for prospective livestock, offering immersive experiences for users to explore various scenarios. Furthermore, the integration of self-improvement mechanisms, such as Reinforcement Learning (RL), elevates the capabilities of DSS by enabling continuous learning and decision improvement over time. RL, a branch of machine learning, empowers DSS to make decisions based on rewards or punishments derived from past experiences. AI-driven precision nutrition systems further customize feed formulations for individual animals or groups based on their distinct nutritional needs, health status, and performance goals. Real-time data from sensors, wearable devices, and automated monitoring systems enable these systems to adjust feed compositions to optimize animal health and performance dynamically. Additionally, AI-driven precision nutrition systems customize feed formulations based on individual animals’ distinct nutritional needs, health status, and performance goals. Real-time data from sensors and wearable devices enable dynamic adjustments to feed compositions, optimizing animal health and performance while minimizing resource waste. AI evaluates nutritional properties, suggests substitutions, and minimizes costs without compromising feed quality, thereby promoting sustainability in feed production [96]. AI-powered monitoring systems continuously oversee feed production processes, ingredient quality, and final product characteristics in real time. By identifying deviations or anomalies, these systems prevent quality issues, reduce waste, and enhance resource efficiency, contributing to sustainable agriculture practices [97]. AI-driven decision support systems bridge the gap between precision livestock farming, sustainable feed formulation, and efficient resource management, paving the way for resilient and environmentally conscious agriculture. Figure 2 illustrates the use of AI decision support systems feed formulation.

## 3. Perspectives

This study contributes to addressing the challenges posed by global population growth in the livestock industry, emphasizing the need to enhance animal productivity and profitability for human food security. Feed producers play a fundamental role by continuously refining formulations to meet evolving livestock needs. We critically explored pressing issues in feed formulation over the decades, particularly highlighting the urgency for environmentally sustainable practices amid climate change challenges. In response to the industry’s dual challenge of meeting global demands and mitigating environmental impacts, the integration of artificial intelligence (AI) is proposed. AI technologies are key in optimizing formulation processes, ensuring compliance with standards, enhancing user experience, and contributing to overall sustainability. We highlight the essential role of AI-driven decision support systems in innovatively addressing the complex demands of animal feed formulation. This work aims to extend the knowledge base and further efforts to establish a resilient and sustainable system through a comprehensive review of current technologies and focus on future trends. The emphasis lies in recognizing the potential of formulation techniques to impact the environment and enhance animal quality and performance positively. We conclude with actionable insights for improving animal feed production by examining and connecting formulation models and decision support tools, as shown in Figure 2. The anticipated outcome is a more informed and sustainable decision-making process, contributing significantly to global efforts in climate change mitigation and environmental stewardship in animal production agriculture.

## 4. Conclusions

In future studies, integrating computer vision technology into feed rationing processes holds immense potential. Computer vision systems can accurately analyze factors such as animal behavior, body condition, and feed consumption patterns to optimize feed-rationing strategies. By leveraging machine learning algorithms, these systems can continuously learn and adapt, ensuring precise and tailored feeding programs for livestock. This approach enhances efficiency, minimizes feed wastage, and promotes better animal health and welfare. Additionally, computer vision technology enables real-time monitoring and feedback, facilitating informed decision-making and driving advancements in sustainable feed formulation practices.

This paper emphasizes the following key points:

1. The livestock industry faces the complex task of meeting global demands while mitigating environmental impacts.
2. AI emerges as a solution to optimize the formulation process and ensure compliance with regulatory standards.
3. AI technologies could elevate the user experience and provide practical solutions for a resilient and sustainable future in animal feed formulation and the overall sustainability of animal agriculture.
4. The core emphasis lies in recognizing the potential of formulation techniques to positively impact the environment and enhance the overall quality and performance of animals.
5. Integrating AI-driven solutions emerges as an innovation, guiding the livestock industry toward a future of efficiency, precision, and sustainability.

## Figures and Tables

**Table 1:** Feed formulation technique and various models used from 1980 to 2020.

**Year**		**Livestock**	**Application/Objective**	**Reference**
1980		Beef	A beef cattle ration formulation technique that satisfies the nutrient standards.	[52]
		Lactating cow/growing cattle	Existence of variability in the nutrient requirements of individual animals and errors in feed intake prediction.	[53]
		Mature dairy cows	The integration of current biological knowledge into mathematical models that can be used both as a framework for defining research priorities and for making decisions on dairy cattle feeding.	[54]
		Unspecified	The introduction of multiple-criteria decision-making techniques to agricultural systems modelers and the demonstration of their use in livestock ration formulation.	[48]
1985		Broilers Chick	Least-cost optimizer. This model calculates the dietary nutrients dynamically for the entire growth period.	[55]
		Laying hens	To simultaneously determine both the optimum economic dose and nutrient density of feed for laying hens.	[56]
1990		Unspecified	The development of a ration formulation program.	[50]
		Turkey	Predicting the values of the flock performance indicators of significant economic importance, body weight and feed consumption.	[57]
		Dairy cow	A method for relaxing the right-hand sides of a least-cost ration.	[58]
		Dairy cow	Minimize cost and maximize the amounts of stored feeds in the diet.	[59]
		Dairy cow	The relaxation of over-rigid specifications of nutrient requirements for livestock rations.	[37]
		Not specified.	To formulate two non-linear optimization problems as an iterative sequence of LP problems.	[60]
		Dairy cattle	Treats the fuzziness of the right-hand side of constraint functions in nutrient requirement. It also combines both relaxation and improvement in trade-offs considered.	[61]
		Dairy cow	The determination of a realistic optimal replacement strategy.	[62]
1995		Dairy cow	An estimate of the cost of feeding a cow was studied, and the identification of alternative lower-cost forage practices with the potential to replace feeding hay.	[63]
2000		Broiler grower ration	To minimize nutrient variance and ration cost.	[38]
		Broiler	Identification of optimal feed mix that maximizes profit margin.	[64]
2005		Pig	Determination of the optimal composition for an animal diet that will provide the best result for the animal.	[5]
		Dairy cows	To operationalize a programming method that can be used on a farm in deciding the optimum diet formulation and minimization of nutrient loading.	[65]
		Sheep, dairy cow, and buffalo	Three models were developed for maximizing animal weight gain in sheep, cow milk yield, and buffalo weight gain.	[66]
		Pig	An optimization method based on the traditional least-cost method that reduces feed cost and total phosphorus.	[67]
		Beef	Optimization of feed formulation with least-cost ration while ensuring a lesser risk of worsening its nutritional value.	[68]
		Unspecified	This method confronts the cost of the ration and meets nutrient requirements while enhancing the feed formulation process.	[69]
		Dairy cow	By applying a penalty function and merging two models, a more efficient ratio was formulated in terms of economic and nutritional values.	[70]
		Pig	A 3-step approach is used to formulate a nutritionally and economically balanced ration that meets organic farming conditions.	[70]
		Beef	Preparation of a user-friendly tool for optimal ration formulation via the combination of mathematical programming techniques.	[70]
		Unspecified	Determination of optimal livestock feed blend.	[71]
		Beef	In the determination of economically and environmentally optimal rations using various feed resources.	[72]
		Dairy cow	A linear fuzzy model was used instead of linear programming models, reducing feed cost to about eight percent.	[73]
		Poultry	The application of a linear programming approach for higher productivity level.	[74]
		Unspecified animal	The development of a feed formula to optimize maximum weight gain.	[75]
		Cattle	The cost minimization of ration and maximization of the value of nutrients at different weight stages.	[30]
2010		Fish feed	The development of an optimized feed formulation model to enhance productivity.	[76]
2020		Broiler	To optimize energy density and bird performance.	[77]
		Dairy cow	To develop the optimal feed mix for dairy cows at different stages of livestock.	[78]
		Livestock	The determination of the optimal livestock feed mix.	[78]
		Broiler	To minimize the total production cost of the diet ration and make it affordable for the average farmer.	[36]
		Sheep, cattle, and rabbit	High priority materials are selected by the decision-makers to solve the mixture problem and cost minimization and optimization was also achieved in the study.	[79]
		Unspecified animal.	Feed formulation technique to produce an optimal solution that is both economical and high energy yield.	[31]
		Broiler	Feed ration formulation to find the optimal quantities of Moringa Oleifera	[80]
		Poultry	Development of an optimal solution that is both economical and brings out the best energy yield.	[79]
		Poultry	The development of a generic decision support system for feed formulation optimization.	[79]
		Beef Cattle	Identifying a least-cost ratio that captures the volatility of feedstuff prices.	[6]
		Dairy cattle during pregnancy	The use of a real-coded genetic algorithm (RGA) to find least-cost feedstuffs without any nutrient deficiency.	[81]
		Animal diet	The evaluation of optimal values for feed components to achieve minimum cost and maximum shelf life.	[82]
		Animal diet	To obtain an optimized animal ration at minimum costs and better shelf life.	[82]
		Poultry	To find the minimum cost of feeding, considering many factors (the purpose of breeding, poultry type, growth stage, nutritional requirements, and available feedstuffs).	[83]
		Broiler	The development of a tool for broiler diet formation designed to target and reduce specific environmental impact categories in the UK and US.	[84]
		Pig	An algorithm was developed to formulate diets that minimize the feed cost per kg live weight gain (least-cost) for the grower/finisher phase in each tax scenario.	[85]
		Dairy Cow	The development of algorithms for formulating an optimal feed mix with minimal cost and maximum shelf life at distinct stages of livestock.	[30]
		Shrimp	To formulate the most appropriate combination of ingredients for shrimp to tackle the feed mix problem by leveraging genetic algorithms.	[86]
		Broiler	To access the nutritional and economic benefits of using a multi-stage linear programming model as an optimization model in broiler feed formulation.	[84]
		Swine	Studied the economic and environmental concerns (cost and phosphorus content) involved with introducing distiller’s dried grains with soluble to swine feed rations, which minimized cost and reduced the phosphorus content.	[87]
		Sheep, cattle, and rabbit	The use of a model that specifies the type of material and the amount of the material to be selected.	[88]
		Non-pregnant dairy buffaloes	Optimization of the total mixed ration cost where the vital requirements for the feed are met with no deficiency in nutrients.	[89]
		Dairy and beef cattle	An evolutionary algorithm minimizes feed cost and deviation from the specified requirements.	[28]
2020		Poultry	The use of linear programming technique in the minimization of cost in a small-scale poultry farm.	[83]
		Poultry	In the determination of economically and environmentally optimal rations using various feed resources.	[90]
Legend				
		Methods/Models		
		Linear programming algorithm		
		Multi-goal programming		
		Multi-objective optimization algorithm		
		Mixed-integer programming		
		Simulation		
		Combination spreadsheet		
		Non-linear programming		
		Multi-criteria decision model		
		Stochastic programming		
		Goal programming		
		Least-cost feed formulation method		
		Linear fuzzy model		
		proximal bundle approach		
		Real-coded genetic algorithm		
		Java Programming		
		Evolutionary algorithm		
		Multi-stage linear programming		

## Data Availability

Data are contained within the article.

## References

[1] Uyeh D.D., Mallipeddi R., Pamulapati T., Park T., Kim J., Woo S., Ha Y. (2018). Interactive Livestock Feed Ration Optimization Using Evolutionary Algorithms. Comput. Electron. Agric..

[2] United Nations (2019). World Population Prospects 2019.

[3] Eeswaran R., Nejadhashemi A.P., Faye A., Min D., Prasad P.V.V., Ciampitti I.A. (2022). Current and Future Challenges and Opportunities for Livestock Farming in West Africa: Perspectives from the Case of Senegal. Agronomy.

[4] Omidiora E.O., Adegoke B.O., Aderounmu G.A. (2013). Review of Livestock Feed Formulation Techniques. J. Biol. Agric. Healthc..

[5] Castrodeza C., Lara P., Peña T. (2005). Multicriteria Fractional Model for Feed Formulation: Economic, Nutritional and Environmental Criteria. Agric. Syst..

[6] Helmuth L.K. (1995). Minimum Cost Beef Backgrounding Ration Utilizing Alternative Feedstuffs & Stochastic Prices. A Thesis Submitted to the Graduate Faculty of North Dakota State University of Agriculture and Applied Science.

[7] Pomar C., Remus A. (2022). 242 The Impact of Feed Formulation and Feeding Methods on Pig and Poultry Production on the Environment. J. Anim. Sci..

[8] Aldoseri A., Al-Khalifa K.N., Hamouda A.M. (2023). Re-Thinking Data Strategy and Integration for Artificial Intelligence: Concepts, Opportunities, and Challenges. Appl. Sci..

[9] Pathumnakul S., Piewthongngam K., Apichottanakul A. (2009). A Neural Network Approach to the Selection of Feed Mix in the Feed Industry. Comput. Electron. Agric..

[10] Amiri Z., Heidari A., Navimipour N.J., Unal M., Mousavi A. (2024). Adventures in Data Analysis: A Systematic Review of Deep Learning Techniques for Pattern Recognition in Cyber-Physical-Social Systems. Multimed. Tools Appl..

[11] Ahmed S.F., Alam M.S.B., Hassan M., Rozbu M.R., Ishtiak T., Rafa N., Mofijur M., Shawkat Ali A.B.M., Gandomi A.H. (2023). Deep Learning Modelling Techniques: Current Progress, Applications, Advantages, and Challenges. Artif. Intell. Rev..

[12] El Chami D., Daccache A., El Moujabber M. (2020). How Can Sustainable Agriculture Increase Climate Resilience? A Systematic Review. Sustainability.

[13] Pesti G.M., Choct M. (2023). The Future of Feed Formulation for Poultry: Toward More Sustainable Production of Meat and Eggs. Anim. Nutr..

[14] Lamine C. (2015). Sustainability and Resilience in Agrifood Systems: Reconnecting Agriculture, Food and the Environment. Sociol. Rural..

[15] Herrero M., Thornton P.K., Gerber P., Reid R.S. (2009). Livestock, Livelihoods and the Environment: Understanding the Trade-Offs. Curr. Opin. Environ. Sustain..

[16] Thornton P.K. (2010). Livestock Production: Recent Trends, Future Prospects. Philos. Trans. R. Soc. B Biol. Sci..

[17] Mupawaenda A.C., Chawatama S., Muvavarirwa P. (2009). Gender Issues in Livestock Production: A Case Study of Zimbabwe. Trop. Anim. Health Prod..

[18] Benton T., Gallani B., Jones C., Lewis K., Tiffin R., Donohoe T. (2012). Severe Weather and UK Food Chain Resilience.

[19] Morgado J.N., Santeramo F., Lamonaca E., Ciliberti M.G., Caroprese M. (2022). Meta-Analysis and Systematic Literature Review of Climate Change Effects on Livestock Welfare. EFSA J..

[20] Xu D., Kai H., Gweyi J.O., Parker M.E., Org M. (2022). The Impact of Climate Change on Food Systems, Diet Quality, Nutrition, and Health Outcomes: A Narrative Review. Front. Clim..

[21] Gauly M., Ammer S. (2020). Review: Challenges for Dairy Cow Production Systems Arising from Climate Changes. Animal.

[22] Angel S., Parent J., Civco D.L., Blei A., Potere D. (2011). The Dimensions of Global Urban Expansion: Estimates and Projections for All Countries, 2000–2050. Prog. Plan..

[23] Webster A.J.F. (2020). Understanding the Dairy Cow.

[24] Gallenti G. The Use of Computer for the Analysis of Input Demand in Farm Management: A Multicriteria Approach to the Diet Problem. Proceedings of the First European Conference for Information Technology in Agriculture.

[25] Rose S.P. (1996). Principles of Poultry Science.

[26] Minson D. (1990). Forage in Ruminant Nutrition.

[27] Underwood E.J. (2022). The Mineral Nutrition of Livestock.

[28] Uyeh D.D., Pamulapati T., Mallipeddi R., Park T., Asem-Hiablie S., Woo S., Kim J., Kim Y., Ha Y. (2019). Precision Animal Feed Formulation: An Evolutionary Multi-Objective Approach. Anim. Feed Sci. Technol..

[29] Saxena1 P., Chandra M. (2012). Animal Diet Formulation Models: A Review (1950–2010).

[30] Saxena D.P., Khanna N. Formulation and Computation of Cattle Feed Mix by Using TORA and LINGO: Minimization of Adverse Effect of Nutrient Ingredient. Proceedings of the 2014 Innovative Applications of Computational Intelligence on Power, Energy and Controls with their impact on Humanity (CIPECH).

[31] Abayomi O.O., Temitope A.K., Oyelayo O., Oluseye A.A. (2015). A Productivity Outlook for a Multi-Criteria Animal Feed Formulation Problem: A Case Study of Nigerian Feed Mill Industry. Int. J. Adv. Technol..

[32] Knapka J.J. (1997). Natural-Ingredient Diet: Managing the Variation in Dietary Nutrient Concentrations. Lab. Anim..

[33] Rahman R.A., Kendall G., Ramli R., Jamari Z., Ku-Mahamud K.R. (2017). Shrimp Feed Formulation via Evolutionary Algorithm with Power Heuristics for Handling Constraints. Complexity.

[34] Afolayan M.O., Afolayan M. (2008). Nigeria Oriented Poultry Feed Formulation Software Requirements. J. Appl. Sci. Res..

[35] Darmon N.F., Briend E.A. (2002). Linear and Non-Linear Programming to Optimize the Nutrient Density of a Production Diet: An Example Based on Diets of Preschool Children in Rural Malawi. Am. J. Clin. Nutr..

[36] Samuel Olugbenga O., Olusegun Abayomi O., Adebimpe Oluseye A., Akinbowale Taiwo T., Waugh F.V. (2015). Optimized Nutrients Diet Formulation of Broiler Poultry Rations in Nigeria Using Linear Programming. J. Nutr. Food Sci..

[37] Lara P., Romero C. (1994). Relaxation of Nutrient Requirements on Livestock Rations through Interactive Multigoal Programming. Agric. Syst..

[38] Zhang F., Roush W.B. (2002). Multiple Objective (Goal) Programming Model for Feed Formulation: An Example for Reducing Nutrient Variation. Poult. Sci..

[39] Auliya Y.A., Fadah I., Zarkasi M., Milniadi A.D., Baihaqi Y. (2021). Implementation of a Decision Support System for Optimizing Feed Rations in the Cattle Fattening Industry. Int. J. Sci. Dev. Res..

[40] Niloofar P., Francis D.P., Lazarova-Molnar S., Mc-Kinney M., Vulpe A., Suciu G., Balanescu M. Modeling and Simulation for Decision Support in Precision Livestock Farming. Proceedings of the 2020 Winter Simulation Conference (WSC).

[41] Mellor D.J., Beausoleil N.J., Littlewood K.E., McLean A.N., McGreevy P.D., Jones B., Wilkins C. (2020). The 2020 Five Domains Model: Including Human–Animal Interactions in Assessments of Animal Welfare. Animals.

[42] Heard J.W., Cohen D.C., Doyle P.T., Wales W.J., Stockdale C.R. (2004). Diet Check—A Tactical Decision Support Tool for Feeding Decisions with Grazing Dairy Cows. Anim. Feed Sci. Technol..

[43] Van Milgen J., Valancogne A., Dubois S., Dourmad J.-Y., Sève B., Noblet J. (2008). InraPorc: A Model and Decision Support Tool for the Nutrition of Growing Pigs. Anim. Feed Sci. Technol..

[44] Dobos R.C., Ashwood A.M., Moore K., Youman M. (2004). A Decision Tool to Help in Feed Planning on Dairy Farms. Environ. Model. Softw..

[45] Dicks L.V., Walsh J., Sutherland W.J. (2014). Organising Evidence for Environmental Management Decisions: A “4S” Hierarchy. Trends Ecol. Evol..

[46] Parker C.G. Decision Support Tools: Barriers to Uptake and Use. Proceedings of the Advances in Applied Biology: Providing Opportunities for Consumers and Producers in the 21st Century.

[47] Rossi V., Salinari F., Poni S., Caffi T., Bettati T. (2014). Addressing the Implementation Problem in Agricultural Decision Support Systems: The Example of Vite.Net^®®^. Comput. Electron. Agric..

[48] Rehman T., Romero C. (1984). Multiple-Criteria Decision-Making Techniques and Their Role in Livestock Ration Formulation. Agric. Syst..

[49] Rose D.C., Sutherland W.J., Parker C., Lobley M., Winter M., Morris C., Twining S., Ffoulkes C., Amano T., Dicks L.V. (2016). Decision Support Tools for Agriculture: Towards Effective Design and Delivery. Agric. Syst..

[50] Black J.R., Hlubik J. (1980). Basics of Computerized Linear Programs for Ration Formulation. J. Dairy Sci..

[51] Lancaster L.M. (1992). The History of the Application of Mathematical Programming to Menu Planning. Eur. J. Oper. Res..

[52] Glen J.J. (1980). A Parametric Programming Method for Beef Cattle Ration Formulation. J. Oper. Res. Soc..

[53] Crabtree J.R. (1982). Interactive Formulation System For Cattle Diets. Agric. Syst..

[54] Assis A.G., France J. (1983). Modelling Dairy Cattle Feeding in the South-East Region of Brazil. Agric. Syst..

[55] Talpaz H., Torre J.R., Sharpe P.J.H., Hurwitz S. (1986). Dynamic Optimization Model for Feeding of Broilers. Agric. Syst..

[56] Kleyn F.J., Gous R.M. (1988). A Mathematical Model for the Formulation of Optimal Amino Acid and Energy Concentrations in Feeds for Laying Hens. Agric. Syst..

[57] Parmar R.S., Diehl K.C., Hulet R.M., Collins E.R. (1989). Simulation of Weight Gain and Feed Consumption of Turkeys. Agric. Syst..

[58] Lara P., Romero C. (1992). An Interactive Multigoal Programming Model for Determining Livestock Rations: An Application to Dairy Cows in Andalusia, Spain. J. Oper. Res. Soc..

[59] Lara P. (1993). Multiple Objective Fractional Programming and Livestock Ration Formulation: A Case Study for Dairy Cow Diets in Spain. Agric. Syst..

[60] Munford A.G. (1996). The Use of Iterative Linear Programming in Practical Applications of Animal Diet Formulation. Math. Comput. Simul..

[61] Mitani K., Nakayama H. (1997). A Multiobjective Diet Planning Support System Using the Satisficing Trade-off Method. J. Multi-Criteria Decis. Anal..

[62] Yates C.M., Rehman T. (1998). A Linear Programming Formulation of the Markovian Decision Process Approach to Modelling the Dairy Replacement Problem. Agric. Syst..

[63] May G.J., Tassell L.W., Waggoner J.W., Smith M.A. (1999). Relative Costs and Feeding Strategies Associated with Winter/Spring Calving. J. Range Manag..

[64] Guevara V.R. (2004). Use of Non-Linear Programming to Optimize Performance Response to Energy Density in Broiler Feed Formulation. Poult. Sci..

[65] Joleen Hadrich C.W., Harsh S. Optimal Livestock Diet Formulation with Farm Environmental Compliance Consequences. Proceedings of the 2005 Agricultural and Applied Economics Association (AAEA) Conferences.

[66] Saxena P. (2006). Application of Non-Linear Programming in the Field of Animal Nutrition: A Problem to Maximize the Weight Gain in Sheep. Natl. Acad. Sci. Lett..

[67] Pomar C., Dubeau F., Létourneau-Montminy M.-P., Boucher C., Julien P.-O. (2007). Reducing Phosphorus Concentration in Pig Diets by Adding an Environmental Objective to the Traditional Feed Formulation Algorithm. Livest. Sci..

[68] Kavčič J.Ž. (2008). Stane Spreadsheet Tool For Least-Cost And Nutrition Balanced Beef Ration Formulation. Acta Agric. Slov. Suppl..

[69] Peña T., Lara P., Castrodeza C. (2009). Multi-Objective Stochastic Programming for Feed Formulation. J. Oper. Res. Soc..

[70] Žgajnar J., Juvančič L., Kavčič S. (2009). Combination of Linear and Weighted Goal Programming with Penalty Function in Optimization of a Daily Dairy Cow Ration. Agric. Econ.–Czech.

[71] Babic Z., Peric T. (2011). Optimization of Livestock Feed Blend by Use of Goal Programming. Int. J. Prod. Econ..

[72] Oishi K., Kumagai H., Hirooka H. (2011). Application of the Modified Feed Formulation to Optimize Economic and Environmental Criteria in Beef Cattle Fattening Systems with Food By-Products. Laboratory of Animal Husbandry Resources, Division of Applied Biosciences, Graduate School of Agriculture, Kyoto University, Sakyo-Ku, Kyoto 606-8502, Japan. Anim. Feed Sci. Technol..

[73] SalooKolayi D.D., Yansari A.T., NasseriTJ S. (2011). Application of Fuzzy Optimization in Diet Formulation. J. Math. Comput. Sci..

[74] Oladokun V., Johnson A. (2012). Feed Formulation Problem in Nigerian Poultry Farms: A Mathematical Programming Approach. Am. J. Sci. Ind. Res..

[75] Pratiksha Saxena P.S., Vinay Pathak V.P., Vinod Kumar V.K. (2012). Programming Technique for Animal Diet Formulation: A Non-Linear Approach. Int. J. Food Sci. Nutr. Eng..

[76] Nath T., Talukdar A. (2014). Linear Programming Technique in Fish Feed Formulation. Int. J. Eng. Trends Technol. (IJETT).

[77] Gonçalves C.A., de Almeida M.A., Faria-Júnior M.J.A., Pinto M.F., Garcia-Neto M. (2015). Accuracy of Nonlinear Formulation of Broiler Diets: Maximizing Profits. Braz. J. Poult. Sci..

[78] Saxena P., Khanna N. (2015). Optimization of Dairy Cattle Feed by Nonlinear Programming. Proceedings of the 2015 2nd International Conference on Computing for Sustainable Global Development (INDIACom).

[79] Abayomi O.O., Adewale A.O., Odianosen E.F., Oyelayo O. (2016). A Multi-Criteria Productivity Analysis for Animal Feed Formulation Problem: A Case Study of Nigerian Feed Mill Industry. Int. J. Innov. Res. Sci. Eng. Technol..

[80] Chagwiza G., Chivuraise C., Gadzirayi C.T. (2016). A Mixed Integer Programming Poultry Feed Ration Optimisation Problem Using the Bat Algorithm. Adv. Agric..

[81] Kuntal R.S., Gupta R., Rajendran D., Patil V. (2016). Application of Real Coded Genetic Algorithm (RGA) to Find Least Cost Feedstuffs for Dairy Cattle during Pregnancy. Asian J. Anim. Vet. Adv..

[82] Saxena P., Singh D., Khanna N. (2016). Stochastic Programming and C-Somga: Animal Ration Formulation. Proceedings of the 2016 International Conference on Computing for Sustainable Global Development (INDIACom).

[83] Mallick P., Muduli K., Biswal J.N., Pumwa J. (2020). Broiler Poultry Feed Cost Optimization Using Linear Programming Technique. J. Oper. Strateg. Plan..

[84] Tallentire C.W., Mackenzie S.G., Kyriazakis I. (2017). Environmental Impact Trade-Offs in Diet Formulation for Broiler Production Systems in the UK and USA. Agric. Syst..

[85] Mackenzie S.G., Wallace M., Kyriazakis I. (2017). Howeffective Can Environmental Taxes Be in Reducing the Environmental Impact of Pig Farming Systems?. Agric. Syst..

[86] Rahman R.A., Ang C.-L., Ramli R. (2010). Investigating Feed Mix Problem Approaches: An Overview and Potential Solution. World Acad. Sci. Eng. Technol..

[87] Matteis M.C., Yu T.E., Boyer C.N., DeLong K.L., Smith J. (2018). Economic and Environmental Implications of Incorporating Distillers’ Dried Grains with Solubles in Feed Rations of Growing and Finishing Swine in Argentina. Int. Food Agribus. Manag. Rev..

[88] Udias A., Pastori M., Dondeynaz C., Moreno C.C., Ali A., Cattaneo L., Cano J. (2018). A Decision Support Tool to Enhance Agricultural Growth in the Mekrou River Basin (West Africa). Comput. Electron. Agric..

[89] Kuntal R.S., Gupta R., Rajendran D., Patil V. (2017). Study of Real-Coded Hybrid Genetic Algorithm (RGA) to Find Least-Cost Ration for Non-Pregnant Dairy Buffaloes. Soft Computing for Problem Solving: SocProS.

[90] Hadrich J., Wolf C., Harsh S. Optimal Livestock Diet Formulation with Farm Environmental Compliance Consequences. Proceedings of the Selected Paper Prepared for Presentation at the American Agricultural Economics Association Annual Meeting.

[91] VandeHaar M.J., Black J.R. (1991). Ration Formulation Using Linear Programming. Vet. Clin. N. Am. Food Anim. Pract..

[92] Rani B.P., Naz F., Magda R. (2022). Artificial Intelligence Solutions Enabling Sustainable Agriculture: A Bibliometric Analysis. PLoS ONE.

[93] Van der Poel A.F.B., Abdollahi M.R., Cheng H., Colovic R., den Hartog L.A., Miladinovic D., Page G., Sijssens K., Smillie J.F., Thomas M. (2020). Future Directions of Animal Feed Technology Research to Meet the Challenges of a Changing World. Anim. Feed Sci. Technol..

[94] Gagliardi G., Cosma A.I.M., Marasco F. (2022). A Decision Support System for Sustainable Agriculture: The Case Study of Coconut Oil Extraction Process. Agronomy.

[95] Wang J., Zhao Y., Perumal B. (2022). Managerial Decision Support System Using an Integrated Model of AI and Big Data Analytics. Ann. Oper. Res..

[96] Yang Q., Steinfeld A., Zimmerman J. Unremarkable AI: Fitting Intelligent Decision Support into Critical, Clinical Decision-Making Processes. Proceedings of the 2019 CHI Conference on Human Factors in Computing Systems.

[97] Pastea D., Draghici D.M., Suciu G., Balanescu M., Iordache G.V., Vintila A.G., Vulpe A., Vochin M., Dragulinescu A.M., Popa C.D. (2021). Decision Support Platform for Intelligent and Sustainable Farming. Proceedings of the Trends and Applications in Information Systems and Technologies.

